# Breaking the aging epigenetic barrier

**DOI:** 10.3389/fcell.2022.943519

**Published:** 2022-08-01

**Authors:** Sweta Sikder, Ganesan Arunkumar, Daniël P. Melters, Yamini Dalal

**Affiliations:** Chromatin Structure and Epigenetic Mechanisms, Laboratory of Receptor Biology and Gene Expression, Center for Cancer Research, NCI, NIH, Bethesda, MD, United States

**Keywords:** senescence, histone variants, chromatin dynamics, cancer, nucleosomes

## Abstract

Aging is an inexorable event occurring universally for all organisms characterized by the progressive loss of cell function. However, less is known about the key events occurring inside the nucleus in the process of aging. The advent of chromosome capture techniques and extensive modern sequencing technologies have illuminated a rather dynamic structure of chromatin inside the nucleus. As cells advance along their life cycle, chromatin condensation states alter which leads to a different epigenetic landscape, correlated with modified gene expression. The exact factors mediating these changes in the chromatin structure and function remain elusive in the context of aging cells. The accumulation of DNA damage, reactive oxygen species and loss of genomic integrity as cells cease to divide can contribute to a tumor stimulating environment. In this review, we focus on genomic and epigenomic changes occurring in an aged cell which can contribute to age-related tumor formation.

## Introduction

**FIGURE 1:**
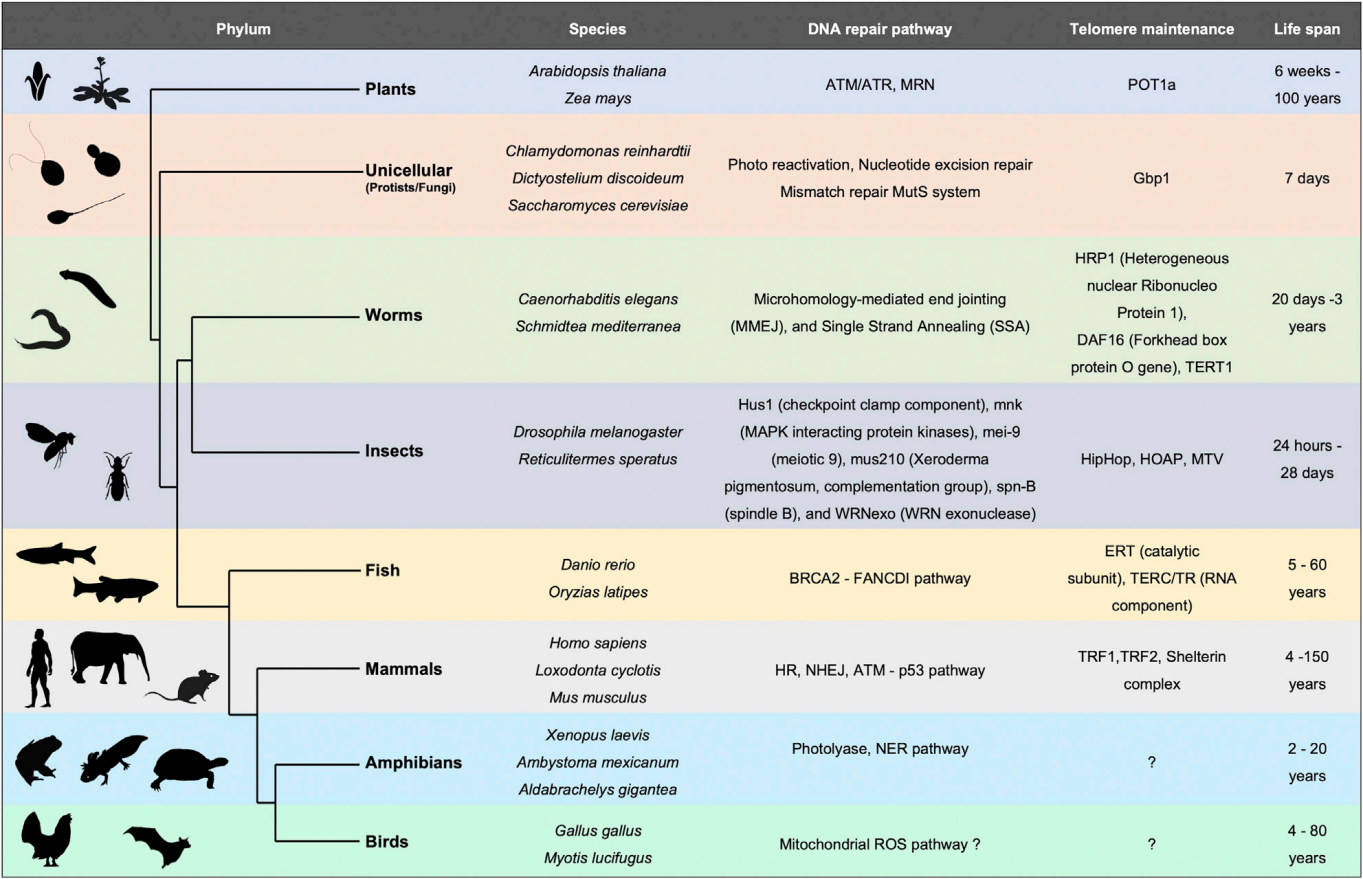
Evolutionary perspective of DNA repair mechanisms and telomere maintenance as primary determinants of lifespan. Table depicting different organisms across the phylogenetic tree with varied lifespans. Summary of DNA repair pathways and telomere associated proteins which positively affect the lifespan.

The mystery of who we are, why we are here, why we age, and die has intrigued the human intellect, spanning art, literature, music, religion, and even the earliest experimental science. For instance, in ancient Egypt, incantations from funerary scrolls of the Book of the Dead dating around 2,323 B.C.E–2,291 B.C.E. were typically painted on the ceiling of tombs with the hope that it would protect and resurrect the deceased in a vividly imagined afterlife. In ancient Mayan culture, the Popul Vuh, one of the last codices to survive the erstwhile Spanish invasion of Mexico, details a cycle of life, death, and eventual resurrection. In the modern scientific era, however, a long-sought-after goal has been to retard or even reverse human aging, by trying to decipher its molecular basis and use chemicals to block or reverse phenotypes associated with aging. This is no trivial feat, as aging is now understood to be a multifactorial process manifested by the gradual decline of physiological functions from the organ all the way down to the cellular, possibly even the molecular level. The phenomenon of functional loss is seen in almost all living organisms ranging from unicellular to multicellular organisms (Figure 1). To gain a deeper understanding of the molecular mechanisms underlying the aging process, several studies have unveiled cellular senescence at a systemic level (Hornsby, 2002; McHugh and Gil, 2018). Cellular senescence was first described almost four decades ago by Hayflick and colleagues, who showed that human cells grown in culture have a finite lifespan (Hayflick, 1965). This finding led to the elucidation of the contrasting effects of cellular senescence. While cell cycle arrest leads to decline of tissue regeneration and repair activity (Hornsby, 2002), it might also serve as a possible tumor-suppressive role by inhibiting cancer cells to proliferate indefinitely (Aunan et al., 2017). Molecular determinants of cellular senescence have established it as a complex phenomenon, as it can be triggered by extrinsic and intrinsic factors, such as radiation or oxidative stress, nutrient deprivation, inflammation, mitogenic signals, progressive telomere shortening, epigenetic changes, chromatin disorganization, perturbed proteostasis, amongst many others (Mikula-Pietrasik et al., 2020). The molecular signatures of each type of senescence are quite diverse, thus altering its functional outcome. To gain further insights, it is important to understand how organisms age across all life forms. For example, what are the common hallmarks, at the organismal, cellular, and molecular levels? Do these events occur in a spatiotemporally uniform fashion or are they random? In this review, we highlight current advances illuminating the causes of cellular senescence specifically in the context of genome organization and genome integrity. We speculate that such nuclear changes may be intricately intertwined with carcinogenesis.

## Evolutionary theories for aging

Lifespan varies tremendously among species ranging from a few hours (bacteria) to thousands of years (sea sponges) (Finch, 1990) (Figure 1). This observation implies a diverse rate of senescence. Fisher (Fisher, 1958), Haldane (Finch, 2010), and later Medawar (Medawar, 1946) proposed that aging occurs when natural selection for fitness traits decreases, or even ceases at a post-reproductive age. This hypothesis further (Medawar, 1946; Medawar, 1952) states that as most organisms die before they reach old age, individuals have a very small probability of being alive and reproductive at an advanced age (Moorad and Promislow, 2010). Consequently, selection primarily occurs in younger generations. A second hypothesis is based on mutation accumulation (Medawar, 1946; Medawar, 1952) states that deleterious mutations within an individual accumulate with age. If these mutations occur after the organism is reproductively active, they will not impact the fitness of the population (Charlesworth, 2001; Hughes and Reynolds, 2005). This is observed in diseases like Huntington’s or Alzheimer’s where the mutation sets on at an older age in humans. A third hypothesis (Williams, 1957) argues for pleiotropic effects of mutation at different ages. For example, a genetic variation could be beneficial early in life when selection is strong, but deleterious late in life when selection is weak. Taking these various modes of evolution into consideration, it would seem unlikely that a single model would suffice to capture diversity in aging mechanisms (or lack thereof) across the species.

Studies across bacteria to mammals have concluded that genome stability is a key factor regulating lifespan (Lidzbarsky et al., 2018) (Figure 1). From bacteria and archaea to eukaryotes, DNA repair mechanisms correlate positively with lifespan (White and Allers, 2018). This sets the stage for DNA-interacting proteins such as replicative/repair polymerases and repair and recombination enzymes, to be implicated in the process of senescence. Unicellular organisms like *E. coli*, the budding yeast *Saccharomyces cerevisiae* or the *Caulobacter crescentus* which undergo asymmetric cell division demonstrate a progressive decline in reproductive potential (Ackermann et al., 2003; Erjavec et al., 2008). Upon deprivation of nutrients, and onset of stress, the bacterial genome undergoes changes. The DNA interacting protein composition also alters dramatically, for example in stationary phase the amount of Dps (DNA binding protein from starved cells) increases, whereas, the highly abundant protein of active genome, FIS, decreases (Ussery et al., 2001). In plants too, DNA repair pathway components as well as organization of the chromatin play an important role in leaf senescence (Guo et al., 2021). Ataxia Telangiectasia Mutated (ATM), suppresses double-strand break-induced expression of senescence-associated transcription factors such as ANAC016, WRKY6, WRKY53, and WRKY75 through histone lysine methylation, thus delaying leaf senescence in *Arabidopsis* (Ay et al., 2009). Although the diversity of proteins involved in maintaining the integrity of DNA among various species is large, understanding how the genome is organized and repaired might hold a cue to the basis of, and potential retardation of the aging process.

## Chromatin structure: An important regulator of aging in eukaryotes

The nucleoprotein complex, chromatin, exists within the limited three-dimensional space of the nucleus (Li and Reinberg, 2011). Chromatin forms a beads-on-a-string structure where each bead is a nucleosome (McGinty and Tan, 2015). Access to nucleosomes on the chromatin fiber is regulated through various epigenetic modifiers such as chromatin remodelers, transcription factors and long noncoding RNAs (Khosraviani et al., 2019). Apart from canonical histones, there are additional histone variants which are incorporated to nucleosomes in a replication-independent manner (Malik and Henikoff, 2003). Incorporation of these histone variants by their chaperones into nucleosome changes not only the physical nature of the nucleosomes, but also lead to the formation of differential chromatin structure either by signaling or by enhanced affinity for additional factors (Kamakaka and Biggins, 2005; Melters et al., 2019). This chromatin variation is found at the centromeres where the canonical histone H3 is replaced by Centromeric protein-A (CENP-A) which helps in assembling the kinetochore and mediating faithful cell segregation (Kamakaka and Biggins, 2005). The importance of histone variants, such as CENP-A, H2A.Z1, and H3.3, are highlighted by their knockout phenotypes which are either embryonic lethal or lead to adverse effects. (Howman et al., 2000; Faast et al., 2001; Tang et al., 2015). Interestingly, all these histone variants have been shown to maintain genomic stability and chromatin integrity (Kamakaka and Biggins, 2005). In the absence of replication-dependent replenishment, does the natural turnover of histones result in gaps in the chromatin fiber that are more susceptible to downstream DNA damage (Lowe et al., 2020)? Conversely, does restoring the epigenetic landscape by overexpressing histone restore genome integrity? It is therefore important to investigate the nucleosomal structure and composition of the aging chromatin.

### Loss of canonical histones over the lifespan of an organism

Due to the finite lifespan of the budding yeast and ease of genetic manipulation and screening, *S*. *cerevisiae* has long served as a model to study eukaryotic aging. In a stunning series of experiments, simply overexpressing core histones in aging yeast cells lengthened their lifespan (Feser et al., 2010). Silent Information Regulator 4 (SIR4) along with SIR2 and SIR3 silence the yeast mating genes and genes in the subtelomere region through heterochromatinization (Kennedy et al., 1995). This SIR-mediated gene silencing is lost as the yeast ages (Khosraviani et al., 2019) suggesting a role of heterochromatin in aging. Furthermore, a gain of function SIR4 mutant was shown to extend its lifespan (Kennedy et al., 1995). Twenty-five years ago, Villeponteau hypothesized that global heterochromatin loss results in aging of mammalian cells including normal human cell lines (Villeponteau, 1997; Tsurumi and Li, 2012; Lee et al., 2020). This hypothesis posits that as cells proceed through successive cell cycles and enter a stage of permanent growth arrest (i.e., replicative senescence), there is a progressive loss of the canonical histones. This histone loss leads to the disruption of heterochromatin at a global scale. This in turn, would lead to perturbation of the transcriptional landscape and expression of previously silenced regions of the genome. In addition to the budding yeast, this concept of chromatin architectural erosion has been documented in organisms like *C. elegans,* Drosophila, mice, and humans (Feser and Tyler, 2011). These studies show a characteristic reduction of repressive histone marks of H3K9me3 and H4K20me3 as well as delocalization of Heterochromatin protein 1 (HP1). In the same vein, overexpression of the heterochromatin binding protein HP1 in fruit flies resulted in a longer lifespan and maintenance of muscle integrity (Larson et al., 2012). In parallel with these model organism findings, the loss of repressive chromatin has also been observed in models of premature aging diseases in humans, such as Hutchinson-Gilford progeria syndrome (HGPS) and Werner syndrome. HGPS patients harbor germline mutations in *lamin A* gene (at chromosome 1q21) resulting in a c-terminal truncated version of the prelamin A called the progerin (Eriksson et al., 2003). The precursor prelamin A contains a carboxyl-terminal cysteine-aliphatic-aliphatic-any amino acid (CAAX) motif which undergoes farnesylation and subsequent cleavage by the zinc metalloprotease ZMPSTE24 (Barrowman et al., 2012). This cleavage results in the formation of mature unfarnesylated lamin A. Mechanistically, the mutation in *lamin A* gene found in HGPS (G608G) activates a cryptic RNA splice donor site, causing an internal deletion of 50 amino acids from prelamin A. This truncated, farnesylated prelamin A variant (progerin) fails to undergo cleavage resulting in accumulation in HGPS patients (Worman and Michaelis, 2018). Studies from cultured cells of HGPS patients replicate features of chronologically aged cells like enlarged nuclei, disorganized nuclear structure, reduction of H3K9me3 and loss of HP1 expression (Scaffidi and Misteli, 2006a; Shumaker et al., 2006). Werner syndrome is another accelerated aging model caused by the mutation in DNA repair gene *wrn.* Mesenchymal cells from mice and human which have been depleted of WRN showed drastic reduction of histone methyltransferase SUV39H1, which plays a vital role in the formation of heterochromatin and its maintenance (Zhang et al., 2015). Thus, global heterochromatin loss holds true for both replicative senescence as well as in premature aging models.

A burning question is: why do histone levels decrease during aging? A study in the replicative senescent IMR90 fibroblasts (obtained from human lung) indicate reduction in Stem Loop Binding Protein (SLBP), which is an important factor regulating histone mRNA stability (O'Sullivan et al., 2010). Genes encoding canonical histones are mostly expressed during S phase of the cell cycle. Transcription of these genes are regulated by the cyclin E-cdk2 mediated phosphorylation of the Nuclear Protein Mapped to the AT locus (NPAT) trans-activator protein (DeRan et al., 2008). As cells divide continuously there is gradual shortening of telomeres resulting in an activated DNA damage response. This in turn, inhibits the phosphorylation of NPAT, thereby decreasing the transcription of histone genes (DeRan et al., 2008). These data point to a surprising connection between sensing of telomere length and homeostasis of core histone genes. A more recent study on chromatin degradation through lysosome mediated pathways also extends this concept of histone loss during senescence (Ivanov et al., 2013).

Despite several studies supporting the general hypothesis of canonical histone degradation over age, there are some contradicting reports that merit closer examination. A recent study in mice tissues obtained from different time points of the mouse lifespan reanalyzed histone H3 occupancy genome wide (Chen et al., 2020). Their results indicate alteration in the H3 occupancy (both increased and decreased) at specific genomic sites, but no profound changes in total H3 expression levels were found. A potential limitation of the study was the use of H3 specific antibody which recognizes all H3 isoforms (variants) rather than specifically recognizing a particular histone H3 variant. This study indicates that histone loss and resultant reduction of heterochromatin in an aging cell is predominantly cell-type and context-specific. A later study demonstrates that histone variant H3.3 may be recruited at novel genomic sites in aged cells resulting in a significant change in combinatorial histone H3 post translational modifications (Tvardovskiy et al., 2017). This study also highlights the crucial role of histone modifications and the epigenetic enzymes in mediating cell fate regulation. Histone modifying enzymes like that of, Histone deacetylase 4 (HDAC4), was shown to be downregulated in both replication-dependent and oncogene-induced senescent cells (Di Giorgio et al., 2020). A follow up study demonstrated the mechanism of the onset of senescence by specific HDAC4/H3K27ac interactions at senescence super-enhancer regions (Di Giorgio et al., 2021). Depletion of histone acetyl transferases p300, have also been shown to delay replicative senescence in fibroblasts by suppressing senescence-related gene expression (Sen et al., 2019). These studies demonstrate the direct modulation of the chromatin state at specific loci through epigenetic enzymes.

Collectively, these studies signify the chromatin regulation through histone variants or their post translational modifications. Are these genomic sites specific, or do they vary in a cell type or tissue specific manner? How does this replacement affect the chromatin structure at the localized sites? Does replacement of canonical histones with their corresponding variants have implications in mediating long range interactions across the genome? It is interesting to consider how an eroding chromatin landscape is rewired in an aged cell, and whether this impacts gene expression.

### Reorganization of chromatin into senescence associated heterochromatin foci

Striking nuclear structures associated with aging are senescence associated heterochromatin foci (SAHF) (Narita et al., 2003; Zhang et al., 2007). Several studies have focused on deciphering the detailed structure of SAHF. The inner core of the SAHF is enriched for H3K9me3, an accepted proxy for constitutive heterochromatin. This is surrounded by the facultative heterochromatin layer denoted by H3K27me3 (Chandra et al., 2012). Furthermore, several architectural proteins like High Mobility Group A (HMGA) are an integral part of SAHF (Narita et al., 2006). Knockdown of HMGA leads to drastic reduction of SAHF in cells, proving its essential role in SAHF formation and maintenance (Chandra et al., 2012). In addition, SAHF is enriched for the histone variant macroH2A but not H3.3.

One could speculate that SAHF might be enriched with histone variants. It is indeed found that macroH2A is associated with the facultative heterochromatin region (H3K9me2) of SAHF (Zhang et al., 2007) indicating that the nucleosomal composition of SAHF might be enriched with histone variants. It has long been anticipated that the histone variant H3.3 might replace the canonical histone H3 in SAHF particularly because its chaperone histone regulator A (HIRA) is essential for the heterochromatin foci formation (Zhang et al., 2005; Ye et al., 2007; Zhang et al., 2007). Surprisingly, a more recent study shows that H3.3 is not enriched in SAHF but is contained at the promyelocytic leukemia nuclear bodies (PML-NBs) with another H3.3 chaperone ATRX/DAXX in both proliferating and oncogene-induced senescent cells (Corpet et al., 2014). These data signify novel functions of not just histone variants, but also their associated chaperones in oncogenic transformation of aged cells. These data also uncover additional functions of other histone variants in senescence apart from formation of heterochromatin foci.

Further studies on histone composition of aged nuclei could be informative to better designate functional roles in regulating the behavior of senescent cells. In this regard, it is interesting to note that mass spectrometric analysis of aged mouse neurons and human postmortem brains reveal an increased H3.3 pool (Maze et al., 2015). The decrease of canonical H2A histones is replaced by H2A.Z and H2A.J both in mouse and human aged tissues (Contrepois et al., 2017; Stefanelli et al., 2018). Apart from the core histones, linker histone H1 variants, which are primarily involved in local and global chromatin condensation and accessibility (Brockers and Schneider, 2019), is understudied during ageing. One preliminary study suggested exclusion of H1 from SAHF relevant to the observation of chromatin decompaction in SAHF (Funayama et al., 2006). Another study on H1 demonstrates increase in H1.0 both at protein and mRNA level in human dermal fibroblasts when aged in culture (Sekeri-Pataryas and Sourlingas, 2007). The essential centromeric histone variant CENP-A, which mediates faithful and accurate cell division, was found to be reduced in human fibroblasts aged *in vitro* (Maehara et al., 2010). CENP-A is downregulated in both ras induced and replicatively senescent human cells. However, the functional consequence of such downregulation remains to be deciphered. Table 1 lists the histones, histone modifiers and associated proteins implicated in aging. It is to be noted here that several studies predict that the main function of the SAHF is to repress proliferative gene expression in an epigenetic fashion. However, the significance of the histone variants and other gene regulatory factors present outside the SAHF remain open avenues for exploration. Does histone variant replacement alter the accessibility of the chromatin structure? Do the modified nucleosomes harbor different histone posttranslational modifications? Does this impact gene expression, three-dimensional folding, replication timing, repair kinetics, or indeed any other aspect of nuclear biology? Investigating these avenues might provide insights into senescent chromatin structure and function.

**TABLE 1:** Histones and Chromatin modifiers which are altered during ageing.

Name	Type	Expression pattern	Model organism	References
Histones				
H3, H4, H2A, H2B	Canonical histones	Loss	Yeast	Feser and Tyler, (2011)
H3	Canonical histone	Loss	*C.elegans*	Faget et al. (2019)
H3, H2A.1, H4	Canonical histone	Loss	Human fibroblasts	Kennedy et al. (1995)
mH2A, H2A.Z, H2A.J	Histone variant	Accumulation	Human, mouse	(Narita et al., 2003; Chandra et al., 2012; Di Giorgio et al., 2020)
H3.3	Histone variant	Accumulation	Human, mouse	(Zhang et al., 2007; Sen et al., 2019)
H1.0	Histone variant	Accumulation	Human fibroblasts	Eriksson et al. (2003)
Chromatin remodelers				
SWI/SNF	ATP chromatin remodeler	Loss results in shorter lifespan	*C.elegans*	Malaquin et al. (2013)
ISW1/ITCH	Chromatin remodeler	Loss results in extended lifespan	Yeast	Kaur et al. (2019)
Histone modifying enzymes				
SET-1	Histone lysine methyltransferase (ASH-2 complex subunit)	Loss	*C.elegans*	Oberdoerffer and Sinclair, (2007)
EZH2	Histone methyltransferase	Loss	Mouse, human fibroblasts	Zhang et al. (2020)
SUV39H1	Histone methyltransferase	Loss	Human and mouse hematopoietic stem cells	Ocampo et al. (2016)
HP1 beta	Heterochromatin associated protein	Accumulation	Mice and primate tissues	Li et al. (2010)

### Organization of senescent associated heterochromatin foci chromatin in a three-dimensional fashion

Multiple chromatin immunoprecipitation studies revealed that the formation of SAHF is induced by the dissociation of constitutive heterochromatin from the nuclear lamina (Chandra et al., 2015; Scaffidi et al., 2006b). Lamin associated domains (LADs) usually consist of heterochromatic regions which interact with the nuclear lamina (Briand and Collas, 2020). During aging there is a gradual degradation of nuclear lamin protein (Lamin B1) which causes the LADs to detach from the nuclear envelope resulting in a redistribution of the heterochromatin from the periphery to the interior. This process might induce the formation of SAHF (Sadaie et al., 2013). Loss of constitutive heterochromatin also leads to decondensation and activation of satellite repeats. This mechanism is referred to as senescence associated distension of satellites (SADS) (Swanson et al., 2013). SADS formation is an early event found in both mouse and human cells and does not require SAHF formation (Short, 2013). It is also fascinating that although centromeric alpha satellite regions decondenses, there is no large-scale change in the classic heterochromatin marks H3K9me3/H3K27me3. These data suggest a distinct higher order chromatin organization at the centromeric regions (Swanson et al., 2015). The implication of loss of constitutive heterochromatin from the nuclear periphery cannot only be attributed to SAHF formation as (HGPS) progeroid cells are devoid of SAHF (Chandra et al., 2015).

**FIGURE 2:**
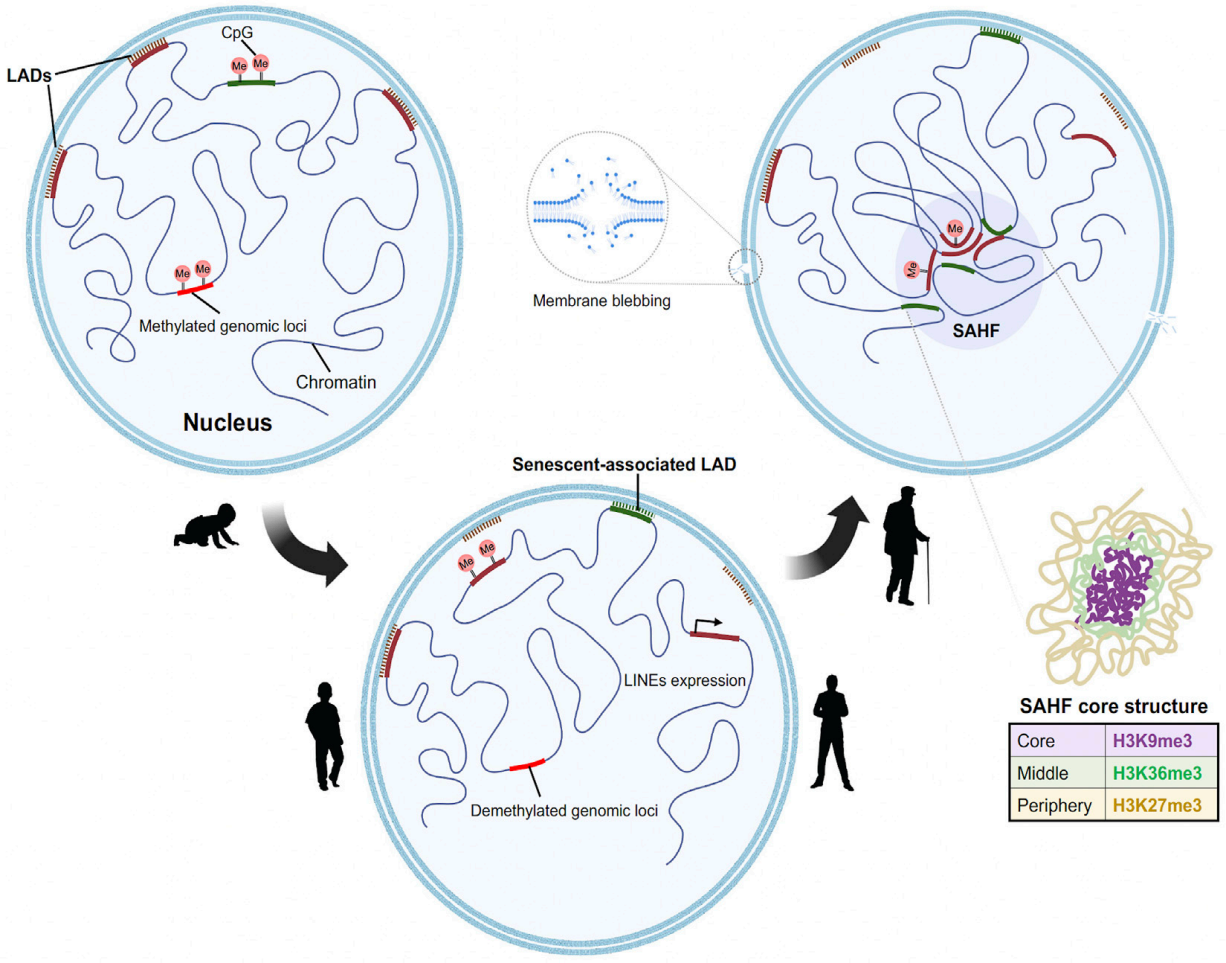
Model for step wise formation of senescence Heterochromatin foci in aged nuclei. In a young proliferating cell (from left)**,** the Lamin associated domains (LADs) (marked in red), consist of compacted H3K9me3 containing constitutive heterochromatin which are tethered to the nuclear lamina. This constitutive heterochromatin is often flanked by H3K27me3 regions. At the onset of senescence (center), the LADs detach first from the lamina which results in the decondensation of the heterochromatin. This process is followed by the spatial clustering of constitutive heterochromatin to form senescent associated heterochromatin foci. Novel regions in the genome gain Lamin B1, moves towards the periphery to form senescent associated LADs. In addition to this there is global DNA methylation changes. Hypomethylation at the LINEs and SINEs activates the elements and leads to aberrant transcription. The genome further undergoes subsequent redistribution to form the senescent foci. The core structure of the SAHF consists of differential chromatin as depicted in layers (right).

One logical speculation is that the reorganization of chromatin in a senescent cell is a two-step process, the decompaction of heterochromatin, followed by the spatial bundling of the decompacted chromatin (Figure 2). The global chromatin organizing factor CTCF, along with cohesin, acts to assemble the higher order chromatin structure. Strikingly, a recent report showed that CTCF is downregulated in aged cells (Hou et al., 2021). Furthermore, in aged cells CTCF DNA binding capacity is impaired upon aberrant transcription of pericentromeric DNA resulting in the expression of senescence associated inflammatory genes examples (Miyata et al., 2021). Taken together, these studies signify the interconnected roles of nuclear architecture, chromatin binding proteins, and region-specific chromatin condensation events in shaping a reformed nuclear landscape during aging.

## Genome integrity and senescence

The process of senescence is prompted by several factors, like accumulation of DNA damage, telomere attrition, epigenetic changes culminating in permanent cell cycle arrest and eventually organismal death. Accumulation of genomic abnormalities during aging can arise due to amassing of unrepaired DNA lesions across the genome owing to the declining quality of repair pathways (Lombard et al., 2005). The term “DNA damage” is quite broad, therefore we categorized them into two main classes based on their origin: endogenous and exogenous DNA damage. Endogenous DNA damage is predominantly caused by replication errors, DNA base mismatches, and formation of topoisomerase-DNA complexes (Chatterjee and Walker, 2017) which are based on cellular enzymatic factors. Reactive oxygen species induce hydrolytic cleavage of the glycosidic bond and deamination of bases (De Bont and van Larebeke, 2004; Alexandrov et al., 2013). Cells have several safeguard mechanisms to repair these lesions. The four major DNA damage repair mechanisms are mentioned as follows. In case of double stranded breaks, cells exploit homologous recombination (HR) or non-homologous end joining (NHEJ) repair depending on the cell cycle stage. Single strand DNA breaks SBs are fixed through the base- or nucleotide excision repair pathways (BER and NER, respectively) whereas mismatched bases are rectified by the mismatch repair (MMR) mechanism (Lindahl, 1976; Pan et al., 2016). Here we discuss the type of DNA damage and disruption of repair pathways which occur in aging and their possible biological significance.

### DNA modifications and reactivation of transposable elements

DNA methylation has been implicated in senescence and acts as a biological clock to determine the progression of age (Horvath, 2013; Weidner et al., 2014). Aberrant DNA methylation is both a signature of extensive hypermethylation and silencing of tumor suppressor genes like that of p21, p16INK4a, and DNA repair genes like BRCA1 which occur in cancer cells (Daniel and Tollefsbol, 2015). Age-dependent hypomethylation of specific long interspersed nuclear element-1 (LINE-1) activates proto-oncogenes such as MET, RAB3IP, and CHRM3 in metastatic colorectal and lung cancer (Hur et al., 2014; Søes et al., 2014). Notably, activation of transposable elements because of DNA hypomethylation and loss of repressive chromatin structure is a common event during aging (Figure 2) (Villeponteau, 1997; Wood and Helfand, 2013). This reactivation could further lead to chromosomal breaks and relocations often characteristic of an oncogenic cell. Collectively these studies suggest a provocative, testable link between non-coding RNA, DNA methylation, altered gene expression and cancer progression. For example, we recently showed that when lncRNA PCAT2 gene is introduced to a naïve chromosome locus it acts in cis to mislocalize centromeric specific histone variant, thus altering the epigenetic memory and chromatin structure at the locus from where it was transcribed (Arunkumar et al., 2022). LncRNAs can also interact with DNA to form RNA–DNA hybrids such as R-loops, to modulate chromatin architecture and accessibility of the transcription machinery to the underlying DNA. Antisense lncRNA TARID forms an R-loop, recognized by growth arrest and DNA damage-inducible-α (GADD45A), at the promoter of tumor suppressor gene TCF21 to trigger local DNA demethylation through TET1 and promote TCF21 gene expression (Arab et al., 2019). Nuclear-abundant lncRNAs NEAT1 and MALAT1 are shown to localize to hundreds of genomic sites in human cells, preferentially to active genes (West et al., 2014). NEAT1 regulates aberrant self-renewal of bone marrow mesenchymal stem cell lineage during skeletal aging by mediating mitochondrial function (Zhang et al., 2022). Whereas, in vascular endothelial cells SIRT6-mediated suppression of MALAT1 resulted in aging-induced endothelial to mesenchymal transition through Snail upregulation (Qin et al., 2019). Therefore, consistent with the role of lncRNAs in the organization of higher-ordered chromatin structure, chromatin-interacting lncRNAs play a major role in the regulation of the chromatin architecture and spatial organization during aging and cancer development. Genome instability through specific DNA or lncRNA mutations then remain a key avenue ripe for exploration.

### A genetic mutational basis for aging

Single nucleotide polymorphisms (SNPs) are the largest source of sequence variation in a DNA sequence among individuals. SNPs act as chromosomal tags and can be used for variations that may be involved in a human disease or disorder. SNP profiling of an individual’s genome helps to study the mechanisms of the aging process as well. Nevertheless, studies showed that mitochondrial DNA (mtDNA) SNPs of individuals reaching a long life, such as the centenarians, are different from that at a younger age (Bessenyei et al., 2004). A longevity-associated mitochondrial genotype, called Mt5178A, that decelerates the frequency of mtDNA mutation in the oocytes, is shown to be present at a higher frequency in individuals reaching a longer life (Kokaze, 2005). Nuclear DNA genotypes as is indicated in the previous section are also associated with aging and related diseases. Studying the frequency of HLA-DR alleles, revealed that allelic distributions were significantly different between control and longevous groups. A high DR13 frequency is commonly seen among both genders in centenarians, whereas males had a higher DR7, and female had a higher DR11 frequency in specific (Ivanova et al., 1998). Expression quantitative trait locus (eQTL) is a genomic locus that associate transcriptomic data sets from an individual to identify gene expression phenotype. A longitudinal twin cohort study using whole-blood gene expression data showed that the expression pattern of subset of genes (2,213) are differential over time (Bryois et al., 2017). This study suggests widespread effects of quantitative trait locus on the transcriptome with an aging signature. In another comprehensive study of 11,672 complex disease-associated SNPs study, Yao et al. (2013) identified 14 sex- and 10 age-interacting eQTLs with significant association. They identified 3 age associated SNPs that have a strong association for SLC44A4 expression due to alternative splicing.

Thus, it is evident that accumulation of mutational events that increase in age could be strongly correlated with gender, ethnicity and ancestorial background. As senescence sets in, the system’s capacity to remove cells carrying altered genotypes is hampered resulting in pathophysiological conditions. Genetic mosaicism arises when balanced or imbalanced chromosomal aberrations such as deletion or duplication occur as cells divide (Lin dstrom et al., 2011; Ma chiela and Chanock, 2017). These altered chromosomes might be formed during early embryonic development or later in life. Thus, these studies indicate a genetic component to aging. However, whether specific DNA sequence alterations result in cell growth inhibition and whether these mutations are enough to potentiate the process of aging remains to be established.

### DNA repair pathway is compromised in ageing tissues

Why do aged cells accumulate mutations? One approach to address this seemingly straightforward question is to compare closely related short- and long-lived animals. A study comparing long-lived and short-lived bats revealed that two genes involved in mismatch repair (MSH2 and MLH1) were significantly reduced in short-lived bats compared to the long-lived ones (Conde-Pérezprina et al., 2012). This indicates that changes in efficiency of DNA repair pathways might contribute to the speed of aging. Furthermore, the study showed that the short-lived bats exhibited increased microsatellite instability with age while the long-lived bats were protected through the expression of enhanced levels of antioxidant enzyme activities (Conde-Pérezprina et al., 2012). Studies from naked mole rats (longest lived rodents with low cancer incidence) also demonstrate similar results in terms of DNA repair activity. A comparative study in mice, naked mole rats, and humans, revealed increased expression of DNA repair genes in humans and naked mole rats that are important for DNA repair pathways such as MMR, NHEJ, and the BER (MacRae et al., 2015). Insights obtained from different DNA repair defective related syndromes demonstrate that defective DNA damage repair pathways lead to premature-aging syndromes (de Boer et al., 2002; Lombard et al., 2005; de Renty and Ellis, 2017). Mice encoding a mutation in DNA helicase gene XPD (trichothiodystrophy (TTD) show premature aging with symptoms such as osteoporosis and cachexia (de Boer et al., 2002). Table 2 lists some of the genetic diseases of DNA repair pathways which exhibit a disrupted aging pattern. Thus, aging of the genome may also be correlated with loss of fidelity or competence in DNA damage repair activity. With a frail repair system, the damaged DNA lesions are uncorrected thus leading to DNA mutations and exit from the cell cycle. Will improving DNA repair activity alone rejuvenate aged cells?

**TABLE 2:** Age-associated disorders which directly affect DNA repair and genome maintenance.

Disease	Genes mutated	Pathway affected	Aging-related symptoms	References
Werner syndrome	WRN	Telomere maintenance, DNA replication and recombination repair	Arthritis, cardiovascular diseases, sarcopenia, atherosclerosis, and increased risk of cancer	Sinclair, (2005)
Bloom syndrome	BLM	DNA replication, recombination	High incidence of cancer, pulmonary disease, diabetes	Ivanova et al. (1998)
XFE progeroid syndrome	ERCC4	ICL, NER	Anemia, cardiovascular and kidney disease, neurodegeneration, sensory loss	Bartkova et al. (2006)
Trichothiodystrophy	TTDA, TTDN1, XPB, XPD, XPG	TC-NER	Bone marrow exhaustion, higher risk of cancer	Bryois et al. (2017)
Ataxia telangiectasia	ATM	DSB repair	Bone marrow exhaustion, diabetes, neurodegeneration	Kosar et al. (2011)
Hutchinson-Guilford progeria syndrome	LMNA	Nuclear lamina function, chromatin architecture	Alopecia, arthritis, cardiovascular diseases, skin aging and atrophy	Eriksson et al. (2003)
Cockayne syndrome	CSA, CSB, XPB, XPD, XPG	TC-NER	Ataxia, cataracts, muscular and neurodegeneration	Hauer and Gasser, (2017)
Fanconi anemia	FANCA-FANCW	ICL	Premature bone marrow exhaustion	Armeev et al. (2021)

ICL, Interstrand DNA crosslinks; TC-NER, Transcription coupled nucleotide excision repair; NER, Nucleotide excision repair.

### Telomere attrition as a source of DNA damage

Apart from the DNA lesions and variations, cellular senescence can be triggered through other mechanisms. An important factor is telomere attrition which is evolutionarily conserved (Figure 1). Telomeres are short tandem repeats of DNA that functions as a protective cap at the ends of the chromosomes to prevent it from double strand breaks at consecutive cell divisions. The Hayflick limit is based on the shortening of telomeres, limiting cellular longevity to a finite number of cell divisions (40–60 for human diploid fibroblast cell lines) (Aunan et al., 2017). This happens due to the low fidelity of DNA polymerases which prevent it from copying the entire sequence at the DNA ends at each cell division subsequently leading to shorter telomere length (Vaiserman and Krasnienkov, 2021). In the mammalian systems, G-quadruplex rich telomeric DNA is more predisposed to oxidative damage compared to other genomic sites (Petersen et al., 1998), and telomere-bound proteins (TRF1 and TRF2) also inhibit DNA repair machinery to access the telomeres (Palm and de Lange, 2008). This prevents the resolution of DNA breaks/lesions, leading to persistent DNA damage signaling stimulated by telomeric DNA (Cesare et al., 2013). In addition to this, recent evidence on the transcriptional events occurring at the telomeres, suggest induction of telomeric dilncRNAs (tdilncRNAs) and telomeric DDRNAs (tDDRNAs) during senescence (Aguado et al., 2020). These non-coding transcripts are essential for the maintenance of DNA damage response activation at dysfunctional telomeres. Sequence specific inhibition of these lncRNAs through antisense oligonucleotides ameliorated the aging effects in HGPS mouse model (Aguado et al., 2019). Furthermore, the DNA damage signaling at the telomeres, can also be prevented by the enzyme telomerase, which is present in limiting amounts in most human somatic cells and in most mammals (Hornsby, 2007). Telomeric chromatin is shown to undergo significant remodeling during aging. Telomerase deficient mice as well as aged human fibroblasts exhibit reduced heterochromatin markers H3K9me3, H4K20me3, and CBX3 at the telomeres, while the euchromatin marker like H3K9ac increases (Benetti et al., 2007). These findings connect the concepts of heterochromatin loss and telomere shortening, signifying that aging is driven by simultaneous endogenous events. The gradual shortening of telomeres can be prevented by the expression of hTERT (telomerase) which is used to immortalize cultured cells (Bodnar et al., 1998) and can be efficiently used for tissue engineering (Shay and Wright, 2000). Despite being an efficient anti-aging therapy, hTERT overexpression has its own shortcomings. A heightened telomerase activity is a signature of cancerous cells (Horn et al., 2013; Jafri et al., 2016). Therefore, further exploration of mechanisms to prevent aging defects is required to prevent its neoplastic transformation.

## Tumor formation in the aging background

The progressive decline of physiological functions during aging results in various pathological conditions including cancer (Yancik, 1997; Berger et al., 2006). Although aging and cancer share some common mechanisms such as disruption of telomere length, genomic instability, diverse epigenetic changes, altered proteostasis, disrupted nutrient sensing and metabolic pathways (Gemble et al., 2015; Gemble et al., 2016; Berben et al., 2021), they lead to divergent cell fates. The process of cellular senescence also plays a crucial role in the process of transformation of an aged cell to malignancy. On the surface, cancer cells and aged cells display conflicting features. Cancer cells are highly proliferative cells, harboring mutations enabling prompt cell division, resulting in high consumption of energy; whereas aged cells accumulate mutations which pose a disadvantage for cell growth and proliferation. However, several studies have associated cellular senescence with cancer development (Berger et al., 2006).

### Genome instability: A common cause of aging and cancer

**FIGURE 3:**
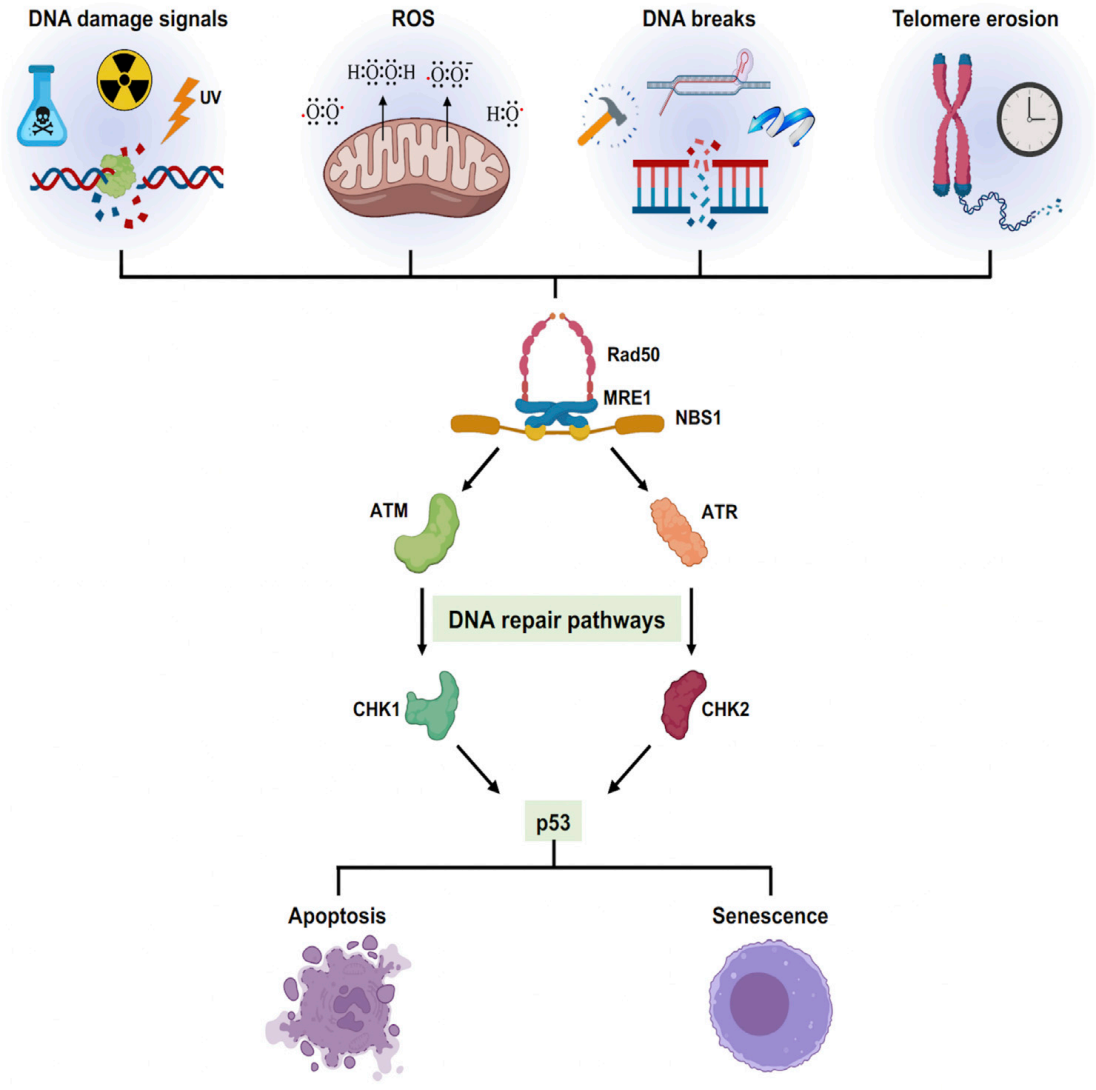
Cellular senescence triggered by different stress signals: Different exogenous and endogenous events trigger a stress response pathway. The stress elicits a DNA damage responsive pathway *via* ATM/ATR converging onto p53 which decides the cellular fate either to cell death or cell growth arrest.

The progressive buildup of DNA damage and mutations are prime drivers of both cancer and aging (Sinclair and Oberdoerffer, 2009). Extensive exposure to endogenous and exogenous DNA damage factors such as ionizing radiation, ultraviolet radiation, tobacco smoking, toxins, reactive electrophiles, alkylating agents, and environmental stress play a significant role in driving genome instability. In mammalian cells, the production of double-strand breaks (DSBs) initiates DNA damage Response (DDR), a global cellular response by triggering checkpoint signaling and DNA repair mechanisms. The MRN (MRE11/RAD50/NBS1) complex binds to double-strand breaks facilitating the activation of ATM signaling to initiate DDR (Uziel et al., 2003). PARP1 and PARP2 are among the first molecules recruited to DNA breaks induced by irradiation as the MRN complex (Haince et al., 2008) followed by γH2AX, an H2A histone variant, accumulation at the damage site and its phosphorylation is amplified by recruitment of MDC1 (Stucki et al., 2005). MDC1 contributes to the recruitment of multiple DNA Damage Response (DDR) pathway members such as RAP80, 53BP1, KAP-1, and BRCA1 (Thompson, 2012). The overall signaling pathway phosphorylates CHK2, p53, and CDC25 to trigger checkpoint activation and cell cycle arrest (Deng, 2006; Huen et al., 2007; Sakasai and Tibbetts, 2008). Remarkably, PARP1 has a dual role, acting as a longevity factor at a younger age, while playing an aging-promoting factor at an older age or in pathophysiological conditions (Haince et al., 2008). Similarly, γH2AX, p53, and BRCA1 are all shown to be involved in aging and related diseases (Mah et al., 2010; Ben-Aharon et al., 2018; Wu and Prives, 2018). Therefore, accumulation of DNA damage caused by both endogenous and exogenous factors over time will promote growth arrest, apoptosis, or cellular senescence (Figure 3). In this context, examining PARP inhibitors and their effect on aging, bears further experimental examination.

### Progressive inactivation of tumor suppressors with age

**FIGURE 4:**
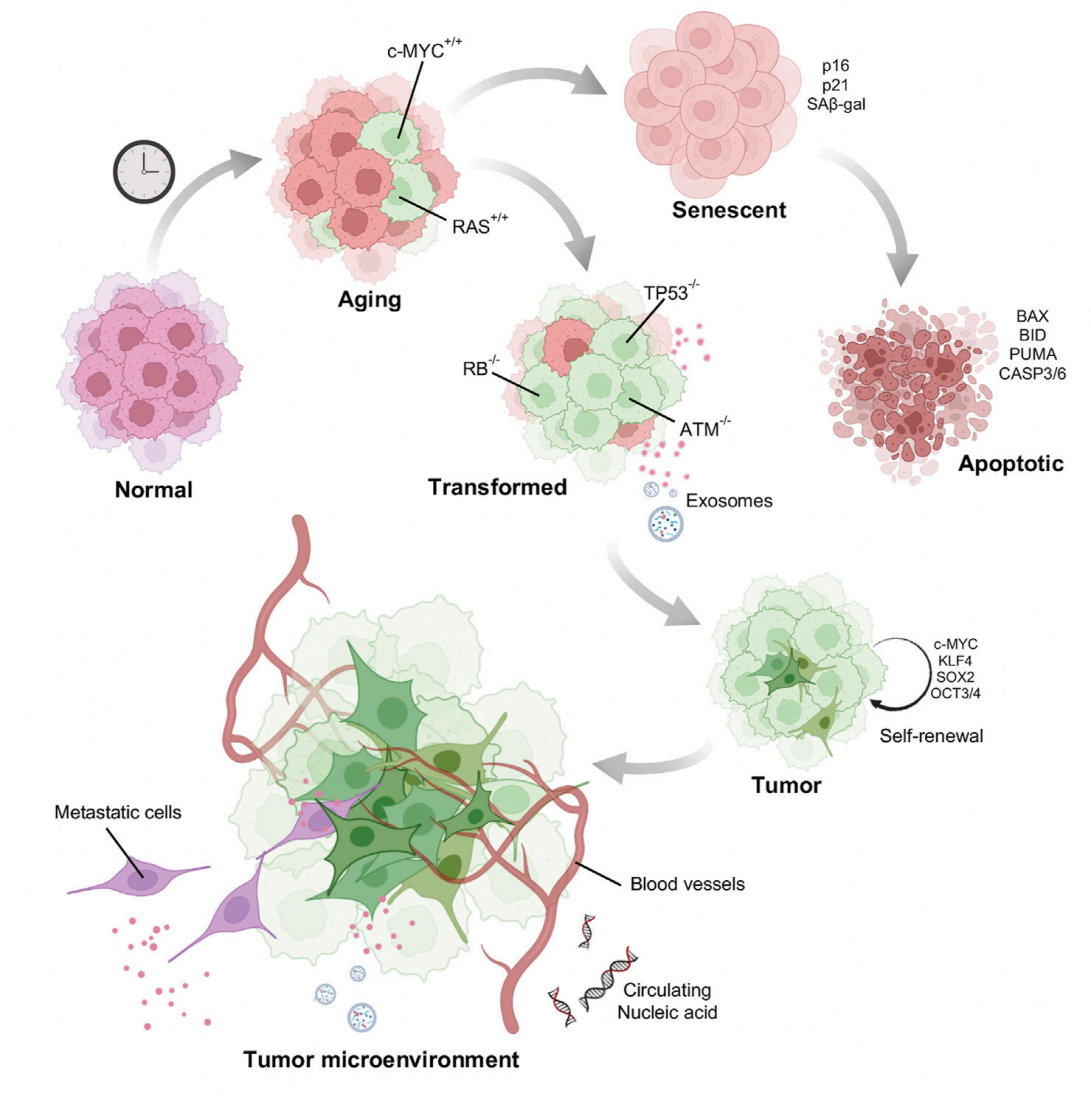
Model of how aged cells potentiate tumor formation: Normal young cells accumulate DNA damage and senesce. A few of the damaged old cells acquire mutations such as activation of oncogenes to induce oncogene induced senescence. Senescent cells after acquiring enormous DNA damage might be directed towards apoptotic pathway. Some cells however, escape death by acquiring other mutations and gain self-renewal property behaving as potential stem cells. Tumor cells once formed is also facilitated by the aging stroma for its growth and metastasis.

Adult stem cells after acquiring enough mutations, epigenetic alterations, depart from the proliferative pool (Sa ntos Franco et al., 2015) (Figure 4). This phenomenon of cell cycle arrest is particularly dependent on p53. The p53 protein encoded by the TP53 gene activates cyclin dependent kinase (CDK) inhibitor p21, which further restricts the activity of CDK4/6 activity. The P16INK4a gene encodes two proteins p14ARF, regulates p53 stability and the p16INK4A protein, an inhibitor of CDK4/6. Thus, both the pathways converge at inactivating the CDK4/6. Inhibition of CDK4/6 activity prevents the phosphorylation of the retinoblastoma protein (pRB). This results in cell cycle arrest at the G1 phase (Ye et al., 2007; McHugh and Gil, 2018). A study by Tyner et al. (2002) compared the propensity of tumor development in wild type, p53 knockout, and mutant p53 (gain of function) background. These data demonstrated high tumor occurrence in p53 knockout mice (Tyner et al., 2002). Mice with a gain of function p53 exhibited very low occurrence of tumor, but fascinatingly, showed signs of premature aging, such as sparse ruffled fur, loss of weight and lethargy. Telomere shortening, another activator of senescence, is also p53 dependent (Chin et al., 1999). Mice carrying extra copies of p53 DNA do not accumulate telomere damage thereby reducing telomere driven aging (García-Ca o et al., 2006). This advantage has also been observed in elephants, which carry extra copies of p53 which is associated with enhanced apoptotic clearance of cells with DNA damage (Seluanov et al., 2018). This response is thought to be a reason why elephants have a low incidence of cancer. All these observations point to the anti-tumorigenic effect of p53 dependent senescence. However, some cells may escape these cellular degradation pathways by acquiring additional strategic mutations allowing them to proliferate even in the presence of an eroded and damaged chromatin landscape. Escaped somatic cells might form a niche in a later stage to develop malignant tumors. The presence of senescent cells in tumor tissues have been reported to arise either spontaneously or through activation of oncogenes (Mikula-Pietrasik et al., 2020).

Chemically induced senescence can be promoted by anti-cancer drugs such as aphidicolin, bleomycin, cisplatin, doxorubicin, etoposide, mitoxantrone, retinols, hydroxyurea, carboplatin combined with docetaxel, and many others (Mikula-Pietrasik et al., 2020). This can be triggered by induction of DNA damage, accumulation of reactive oxygen species or by inhibition of DNA polymerases (Ewald et al., 2010). Although chemically induced senescence might have severe side-effects as it can promote cancer cell proliferation (Alspach et al., 2013). Induction of senescence, particularly by the inactivation of certain tumor suppressors like that of SHP2 (Serrano, 2015) and PTEN (Toso et al., 2014) also facilitate tumor growth. These studies propose a two-hit hypothesis for cancer development from aged cells. Like one bad apple in a basket which spoils the whole lot, a logical question to ask is whether an aged cell acquiring an oncogenic mutation could potentiate tumor cell population? In other words, does the aging cell provide a favorable microenvironment for tumor growth?

### Aged microenvironment favors tumor growth

In the last two decades there have been studies trying to decipher the molecular mechanism of pro-oncogenic activity of senescence. An important factor might be the formation of an immunosuppressive tissue microenvironment. Senescent cells elicit a secretory phenotype called SASP (senescent associated secretory phenotype), characterized by an overproduction of a variety of chemokines, growth factors (EGF, bGF, VEGF, and TGF-β1), cytokines along with several extracellular matrix constituents and remodeling proteins (fibronectin, collagens, laminin, MMP-1, −3). Apart from causing major chronic inflammation, SASP also act *via* autocrine and paracrine pathways (Acosta et al., 2008; Acosta et al., 2013). This enables SASP to inhibit cell growth and promote senescence spreading to distant healthy bystander cells. The proteins such as IL-6, IL-8 (Kojima et al., 2013; Ortiz-Montero et al., 2017), MMP-1 have been shown to induce the paracrine responses and play an active role in tumor progression and metastasis (Faget et al., 2019). Replicative senescent skin fibroblasts secreting MMP-1 and MMP-2 displayed activation of PAR-1 in tumorigenic keratinocytes and enhanced their invasive activity (Malaquin et al., 2013). Aged human skin fibroblast expresses reduced levels of hyaluronan and proteoglycan link protein 1 (HAPLN1) leading to a more organized ECM, which promotes the metastasis of melanoma cells (Kaur et al., 2019) (Figure 4).

## Conclusion and perspectives

Comprehending the role of molecular processes such as DNA damage repair, telomere shortening, nuclear (Oberdoerffer and Sinclair, 2007) and chromatin changes along with epigenetic alterations which drive aging as well as aging related diseases may hold a key to the “elixir of life.” Of late the resurrection of aged cells back to cellular proliferation has garnered attention from various molecular biologists. The use of Yamanaka factors (OCT4, NANOG, SOX2, KLF4, and MYC) reprograms cells to a partially undifferentiated stage which is shown to ameliorate some of the functions of aged fibroblasts as well as fibroblasts obtained from progeroid models (Zhang et al., 2020). The transient expression of these factors rescued the levels of H3K9me3 and DNA damage marks such as γ-H2AX (Ocampo et al., 2016). Caloric restriction is another mode of rejuvenation of aged cells extensively studied in mice and human cells (Sinclair, 2005; Li et al., 2010). Interestingly, this mode of rejuvenation also acts through the epigenome. Glucose restriction induced increase in H3K4me2 at hTERT and H3K9me3 at the p16INK4a promoter, respectively which accelerated cellular lifespan by activation of hTERT and repression of p16INK4a (Li et al., 2010). All these studies fortify the beneficial role of heterochromatin in protecting the genome from DNA damage and neoplastic transformation.

However, there remain several uncharted domains: Is heterochromatin alone sufficient to extend lifespan? Is the reorganization of the heterochromatin guided by the changed DNA methylome in aged cells? A varied number of histone variants are expressed inside as well as outside of the SAHF. What directs them to their specific genomic location upon senescence? The complexity and confusion arise as cells induced by different stress mediated pathways show different epigenetic signatures or varied chromatin organization. Senescent cells found in the pre-cancerous lesions exhibit increased levels of heterochromatic histone modifications (H3K9me2/3 and HP1γ) (Bartkova et al., 2006) but lack in SAHF (Kosar et al., 2011). This discrepancy might be due to the variation in the extent of heterochromatinization of the genome.

We posit that analyzing the biophysical and mechanical nature of aged chromatin polymer in different cell types might provide clues to its natural decay and dysfunction. Despite current technological challenges, even elucidating the half-life or turnover of chromatin factors, including post-translational modifications of nucleosomes, repair factors, chromatin remodelers could be an important start. Knowing these parameters, we can better understand and potentially model how the nuclear landscape changes as cells age. How do these different half-lives impact protein-complex composition and functional stability of transcriptionally active and inactive regions of the genome? Genomic regions which exhibit distinct functions such as promoter, enhancers, and constitutive heterochromatin are marked by the presence of histone PTMs (post translational modifications). Ideally, established histone PTMs are maintained to continue the faithful expression of tissue-specific genes. Rare and unconventional PTMs, such as glypiation, neddylation, siderophorylation, AMPylation, and cholesterolysation, are expected to accumulate in senescent cells, purely by change, or chance, acting as driver epi-mutations. These PTMs influence protein structure and function (Basak et al., 2016). DNA damage also increase histone degradation (Hauer and Gasser, 2017) and histone tail cleavage has been associated with various cellular processes (Yi and Kim 2018). All-atom computational modeling shows that histone tail dynamics modulate the DNA accessibility (Armeev et al., 2021; Peng et al., 2021) and DNA methylation leads to more curved under-twisted DNA (Li et al., 2022). Taking these factors into account, one might predict dramatic differences between the biophysical properties of aged chromatin versus young chromatin. Exploring how material properties of nucleosomes impact the functional outcome of a chromatin fiber and how these properties age, we argue, is the next frontier of chromatin biology. Indeed, development of experimental approaches to circumvent the limitations of time and optimizations of methods are required to study aging chromatin in a microfuge tube.

Finally, the study of aging needs to be expanded from murine/human cells to that of long-lived whales, termites or even plants which live for hundreds of years. These species may provide unanticipated and novel mechanisms of aging and rejuvenation (Holtze et al., 2021) that have eluded our own species. Will the secret “ambrosia of Greek Gods” be found in the genomes of more age-resilient species, such as that of the humble tardigrade (Hashimoto et al., 2016)? It is remarkable that the 21st century thus far has been marked by devastation caused by nano-pathogens and non-pathogenic climate extremes. To quote from a recent novella by the brilliant sci-fi writer Ted Chiang *“*Four things do not come back: the spoken word, the sped arrow, the past life, and the neglected opportunity.” Billionaire Trekkie space pioneers compete with each other, to boldly go where no human has gone before, in the hopes of terraforming distant planets. Examining how human lifespan and aging impacts our potential for exploration is now no longer in the arena of futuristic sci-fi, but an opportunity for nano-exploration rooted firmly to our species’ survival on this planet.
