# Erratum: Breaking the aging epigenetic barrier

**DOI:** 10.3389/fcell.2022.1027162

**Published:** 2022-09-13

**Authors:** Frontiers Production Office

**Affiliations:** Frontiers Media SA, Lausanne, Switzerland

**Keywords:** senescence, histone variants, chromatin dynamics, cancer, nucleosomes

Due to a production error, the umlaut in author Daniël P. Melters’ name was omitted. The publisher apologizes for this mistake.

The original version of this article has been updated.

